# Effects of cognitive dual-tasking on biomechanics and muscle activity during gait initiation and sit-to-walk in young adults

**DOI:** 10.1371/journal.pone.0328677

**Published:** 2025-07-21

**Authors:** Angeloh Stout, Kaye Mabbun, Ke’Vaughn Waldon, Marvin Alvarez, Stacy Nguyen, Miguel Barcellano, Sandra Cuenca, Chuan-Fa Tang, Aanand D. Naik, Gu Eon Kang

**Affiliations:** 1 Department of Bioengineering, University of Texas at Dallas, Richardson, Texas, United States of America; 2 Department of Mathematical Sciences, University of Texas at Dallas, Richardson, Texas, United States of America; 3 Department of Management, Policy and Community Health, School of Public Health, University of Texas Health Science Center at Houston, Houston, Texas, United States of America; 4 Joan and Stanford Alexander Division of Geriatrics and Palliative Medicine, McGovern Medical School, University of Texas Health Science Center at Houston, Houston, Texas, United States of America; 5 Center for Innovation in Quality, Effectiveness, and Safety, Michael E. DeBakey Veterans Affairs Medical Center, Houston, Texas, United States of America; 6 Institute on Aging, University of Texas Health Science Center at Houston, Houston, Texas, United States of America; Università degli Studi di Milano: Universita degli Studi di Milano, ITALY

## Abstract

The purpose of this study was to investigate the effects of cognitive dual-tasking on gait initiation and sit-to-walk. Twenty-eight healthy young adults performed gait initiation and sit-to-walk under two conditions: while engaging in serial subtraction (dual-task) and without any additional task (single-task). Motion data were collected using a 10-camera optoelectronic motion capture system, synchronized with force plates and surface electromyography. We analyzed spatiotemporal parameters, center of mass displacement, center of pressure trajectory, and lower limb muscle activation patterns. We found that dual-task conditions significantly affected both gait initiation and sit-to-walk patterns, increasing the duration of transitions and mediolateral center of mass displacements, while reducing vertical center of mass displacements and forward propulsion. We also observed a more constrained and less efficient center of pressure path, with reduced posterior displacement during the weight shift phase. Muscle activation, particularly in the tibialis anterior and biceps femoris, decreased during dual-task conditions, indicating altered neuromuscular strategies. These findings suggest a shift in postural control demands and motor performance during dual-task transitions.

## Introduction

Transition to walking from a static position, such as standing posture, i.e., gait initiation (GI), or sitting posture, i.e., sit-to-walk (STW), are essential tasks in daily living. Young adults make 150–400 transitions to walking from any posture daily [1] and older adults may make up to 70 STW daily [2]. Compared to steady-state walking, GI and STW are thought to require a higher level of postural control [3]. For example, in young adults, mediolateral center-of-mass (CoM) displacement is approximately 5 cm during GI [4] and about 6 cm during STW [5], which is more than 1.5 times greater than during steady-state walking (approximately 3 cm) [1].

The greater degree of mediolateral CoM displacement during GI and STW suggests that biomechanical and neuromuscular characteristics of these transitional movements may be associated with mobility impairments and an increased risk of falls. For instance, it was reported that an increased duration of GI is an independent risk factor for multiple falls in older adults [6]. Additionally, older adults with a history of falls have shorter step lengths and greater step length variability during GI compared to those without a fall history [7]. More recently, it was reported that older adults with sensory loss show more unstable GI compared to healthy older adults, with the differences being more severe during GI than steady-state walking [8].

Far fewer studies have focused on STW, but some relevant literature exists. For example, older adults with fear of falling had shorter step lengths and wider step widths during STW compared to those without fear of falling [9]. Furthermore, older adults with a history of falls have less forward momentum during STW compared to those without a history of falls [10]. Although previous GI and STW studies have successfully identified characteristic biomechanical patterns associated with mobility impairments and the risk of falls, many have focused on the tasks themselves under single-task conditions. In more real-life situations, GI and STW tasks are often performed concurrently with another task, known as the dual-task condition.

The dual-task paradigm involves performing two tasks simultaneously, typically comprising a primary task and a secondary task [11]. In human movement research, the primary task often involves a movement of interest, such as walking, while the secondary task may involve unrelated cognitive processing, such as solving mathematical problems, “cognitive dual-tasking.” Researchers frequently test performance under two conditions: the primary task alone (single-task condition) and the primary task combined with the secondary task (dual-task condition). Since even single-task conditions in human movement involve some level of cognitive processing, like attention, working memory, and inhibitory control for safe ambulation [12], adding a cognitive task challenges the primary movement task and may better reflect real-life situations, even in experimental settings. As such, the dual-task paradigm in human movement research has been of great interest for understanding the fundamental motor-cognition interplay, and extensive research has been conducted on age-related mobility declines, early signs of mild cognitive impairment, and the underlying mechanisms of falls in older adults.

Nevertheless, considerable gaps remain in our understanding of the effects of cognitive dual-tasking on human movement. Firstly, most existing dual-task studies have primarily focused on biomechanical and neuromuscular patterns during steady-state walking. While these findings have important implications, there is a notable lack of research examining the effects of dual-tasking on transitional movements such as GI and STW. Because these transitions may require a higher level of postural control than steady-state walking, as evidenced by mediolateral CoM displacement, understanding how a concurrent cognitive task affects GI or STW would provide critical insights into the motor-cognition interplay. Secondly, while a limited number of studies have explored the effects of cognitive dual-tasking on GI in individuals with neurological disorders and older adults [13], there is a notable dearth of research focusing on STW. Given the daily frequency of STW [2] and the potentially different biomechanical and neuromuscular mechanisms involved in transitioning from a sitting posture compared to a standing posture, identifying how cognitive dual-tasking alters STW may provide crucial insights and substantially expand our current knowledge of motor-cognition interplay. Furthermore, although data from individuals with mobility impairments is critically important in human movement research, data from young adults is also vital because young adults can serve as crucial benchmarks, providing reference values to understand the typical functioning of the cognitive-motor system [14].

Accordingly, the purpose of this study was to investigate the effects of cognitive dual-tasking on GI and STW in young adults. We examined how biomechanics and muscle activity are altered during GI and STW under dual-task conditions compared to single-task conditions. We analyzed changes in spatiotemporal parameters, kinematics, kinetics, and muscle activation patterns in response to dual-tasking. We hypothesized that cognitive dual-tasking will significantly change both GI and STW, compared to single-task conditions.

## Methods

### Experimental procedures

University of Texas at Dallas Institutional Review Board Approval #: IRB-22–569.

Written consent was obtained from all participants. Initial approval was granted on August 2, 2022, with a renewal on July 18, 2023. We recruited 28 young adults from the University of Texas at Dallas campus between August 11, 2022 and November 9, 2023. Participants had no history of neurological or musculoskeletal disorders. We used a ten-camera optoelectronic 3D motion capture system (Vicon, Oxford, UK), synchronized with two force plates (Kistler, Winterthur, Switzerland). The cameras circled around a 10-m walkway and collected position data from reflective markers at a sampling rate of 100 Hz. The two force plates were positioned at the start of the walkway and collected the ground reaction force (GRF) and center-of-pressure (COFP) displacement data at a sampling rate of 1000 Hz. We also used wireless surface electromyography (EMG) sensors (Delsys, Natick, MA, USA) to collect data for lower limb muscle activity at a sampling rate of 2000 Hz.

**Fig 1:**
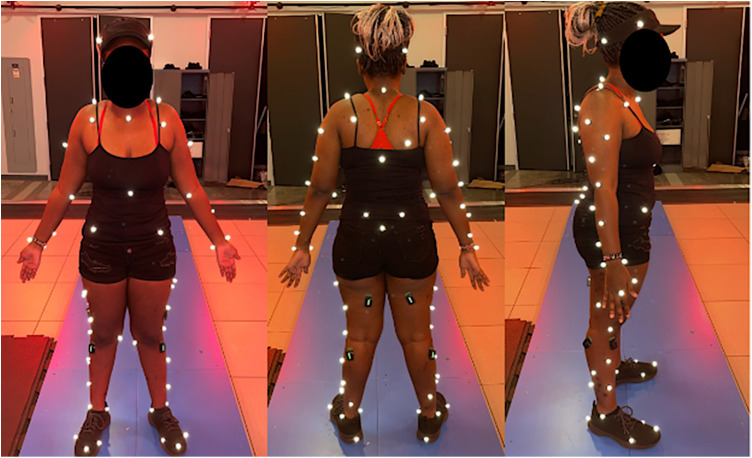
Marker positions. Markers were attached to anatomical landmarks across the entire body.

We collected the participants’ demographic information. Then, participants changed into tight-fitting clothing. We attached 8 EMG sensors for the vastus lateralis, bicep femoris, gastrocnemius, and tibialis anterior. We collected EMG data from maximum voluntary contraction (MVC) trials to normalize muscle activity [15]. For the MVC trials, we instructed the participant to rest for 3 seconds. Next, they were asked to conduct an isometric contraction of the muscle over 2 seconds. They were then instructed to exert maximum strength for 4 seconds, and finally, to relax for 2 seconds. We collected two MVC trials for each muscle. For the vastus lateralis, participants were sitting down with their legs angled at 90 degrees and their ankles were restricted as they performed a unilateral leg extension forward. With the biceps femoris, the participant’s legs began slightly suspended in the air as they were asked to curl their leg posteriorly. For the tibialis anterior, participants performed a toe raise. For the gastrocnemius participants did a standing calf extension. After the MVC trials, we attached 74 reflective markers on participants’ anatomical landmarks, displayed in Fig 1 [16,17].

Participants conducted a static trial. Following the static trial, the medial markers of the elbows, knees and ankles were removed. We collected the participant’s body weight using one force plate. Movement trials began on the 10-meter walkway. Participants performed gait and STW under two single-task and dual-task conditions. For gait trials, participants stood quietly at the start of the walkway on two force plates, with each foot on a separate force plate. They then performed single-task (no concurrent task) and dual-task (with a concurrent cognitive task) gait trials at their self-selected speed. Dual-task trials consisted of a serial subtraction of 3 [18]. In STW trials, participants began seated on an armless and backless chair (height: 0.45 meter) with each foot on a separate force plate. Then we asked participants to walk out straight from the chair at self-selected speed. STW trials were performed in the same single-task and dual-task conditions. Trials were repeated to collect three trials for each condition.

### Data analysis

**Fig 2:**
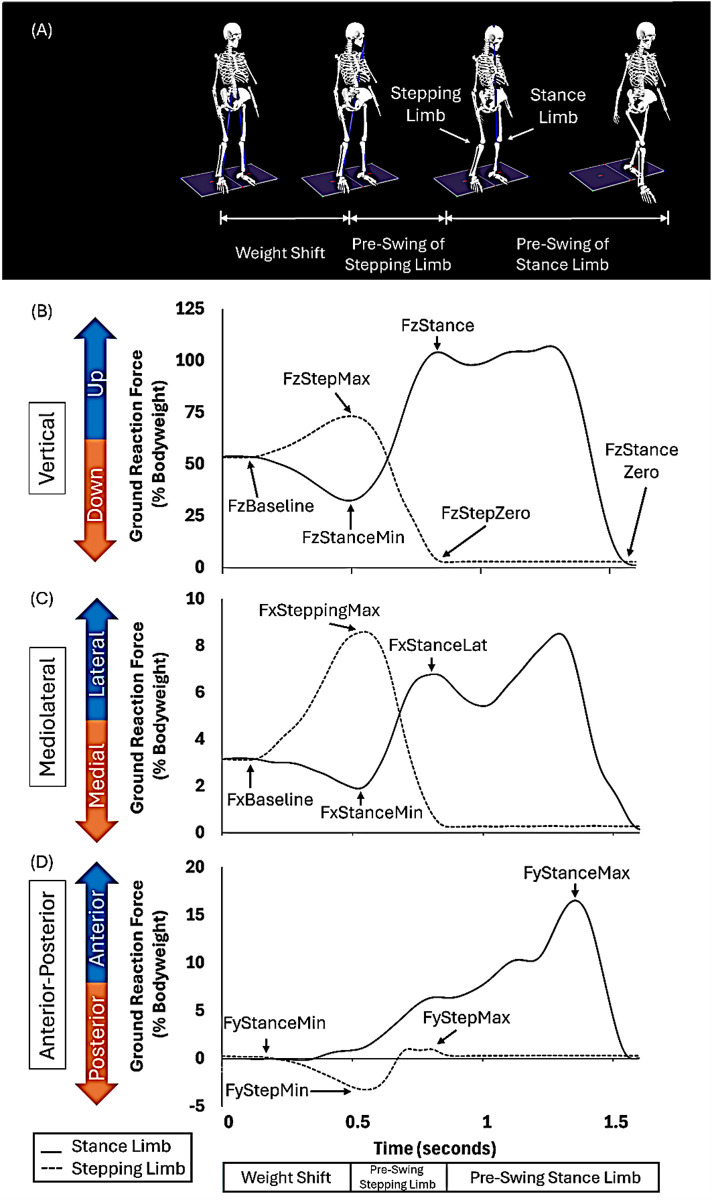
Phases of GI. (A) GI postural characteristics. (B) Vertical GRF of the stepping limb (dotted line) and stance limb (solid line). (C) Mediolateral GRF of the stepping limb and stance limb. (D) Anterior-posterior GRF of the stepping limb and stance limb.

We used Visual3D (C-Motion, Germantown, MD, USA) to calculate spatiotemporal, kinematic, and kinetic variables for GI and STW (Fig 2A). A custom MATLAB (MathWorks, Natick, MA, USA) script was utilized to analyze the EMG data.

We determined GI phases using vertical GRF normalized to body weight (BW) [19]. The weight shift phase was calculated from the start of GI to the weight shift peak (Fig 2B). The start of GI was defined as the instant when the vertical GRF of the stance limb changed by more than three times the standard deviation from the mean vertical GRF during standing. The weight shift peak was defined as the instant of the first local maxima of the stepping limb (FzStepMax), which was equivalent to the first local minima of the stance limb (FzStanceMin). The pre-swing phase of the stepping limb was calculated as the period from the weight shift peak to the toe off of the stepping limb (FzStepZero). The pre-swing phase of the stance limb was calculated from the stepping limb toe off (FzStepZero) to the stance limb toe off (FzStanceZero). The total GI duration was from the start of GI to the stance limb toe off. We calculated the peak CoM displacement in the vertical and mediolateral directions.

In terms of vertical GRF (Fig 2B), we calculated the weight shift stepping max, calculated as the difference between FzStepMax and FzBaseline in GRF values. We calculated the weight shift stance min, defined as the difference between FzStanceMin and FzBaseline in GRF values. We calculated the stance loading, defined as the difference between FzStance and FzStanceMin in GRF values. In terms of mediolateral GRF (Fig 2C), we calculated the stepping lateral, defined as the difference between FxSteppingMax and Fxbaseline in GRF values. We calculated the stance medial, defined as the difference between FxStanceMin and FxBaseline in GRF values. We calculated the stance lateral, defined as the difference between FxStanceLat and FxStanceMin in GRF values. In the anterior-posterior direction (Fig 2D), we calculated the stance propulsion, defined as the difference between FyStanceMax and FyStanceMin in GRF values. We calculated the stepping propulsion, defined as the difference between FyStepMax and FyStepMin in GRF values.

**Fig 3:**
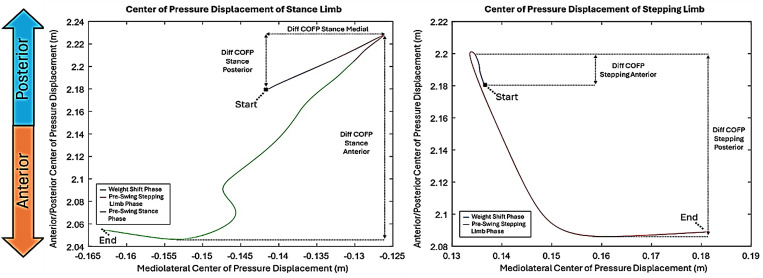
COFP displacement of the stance (left) and stepping (right) limb for GI.

We also calculated COFP displacement of the stance limb and the stepping limb in the anterior-posterior direction (Fig 3).

EMG data were normalized to MVC trials [20]. We filtered the EMG signal by applying a detrend function to eliminate any potential direct current offset. This was followed by a 4^th^ order bandpass Butterworth filter with a cutoff frequency range of 30–300 Hz. This frequency range was selected to capture the physiological spectrum of muscle activation while minimizing low frequency motion artifacts and high frequency noise commonly present in gait data [20]. The filtered signal was then rectified and smoothed using a 2^nd^ order lowpass critical damping filter with a cutoff frequency of 15 Hz to generate the EMG linear envelope. The 15 Hz cutoff smooths the signal while preserving the timing of muscle activation during movement. This filtering process was applied to both MVC and gait trials. To determine the maximum MVC, we computed a moving mean average over 20 frames and identified the peak value as the maximum MVC. The highest value from two MVC trials were used for normalization. We normalized the data by taking the filtered dynamic EMG data and dividing it by the maximum MVC (%MVC). We calculated mean EMG each GI phase and the entire GI.

**Fig 4:**
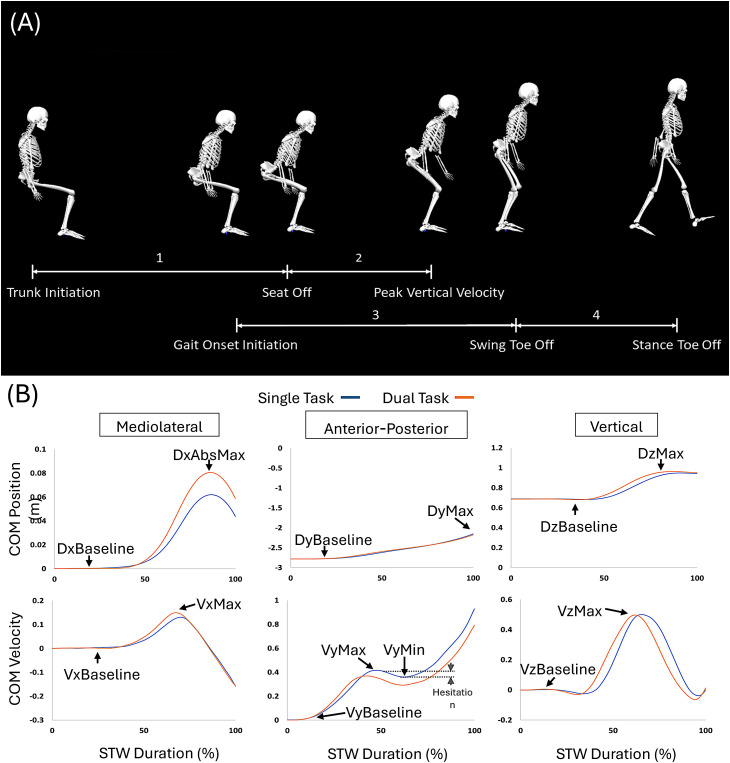
STW Characteristics. (A) STW Phases. (B) CoM position (first row) and velocity (second row) during STW averaged across all participants.

The phases of STW were derived using marker and GRF data [21]. STW was divided into four phases (Fig 4A): (1) the flexion momentum phase; (2) the extension phase; (3) the unloading phase; and (4) the stance phase.

We calculated the total STW duration, and phase durations. Due to the overlap between phases 2 and 3, the normalized phase durations did not equal 100%. We calculated the peak CoM velocity (VxPeak; VyPeak; VzPeak = VzMax – VzBaseline) in all 3 directions (Fig 4B). We calculated CoM displacements in the anterior-posterior (DyMax – DyBaseline) and mediolateral (DxAbsMax – DxBaseline) directions. We also calculated CoM velocity drop in the forward direction (VyMax – VyMin). We calculate the ratio of this drop to the initial forward peak velocity (i.e., Hesitation = Absolute velocity drop/VyMax × 100) [22]. We calculated the initial step length. We calculated the mean EMG in each STW phase and across the entire STW.

### Statistical analysis

Data was analyzed using SPSS 29 (IBM, Armonk, New York, USA). Data was tested for normality via Shapiro-Wilks test. Data that was not normalized was transformed via logarithmic transformation. However, if transformation was non-normalized, a Kruskal-Wallis test was used due to its condition of not assuming normality. A two tailed paired t-test was performed on the normalized data. For all statistical comparisons, we considered p* *< 0.05 as statistical significance. Additionally, we evaluated the effect size as Cohen’s D.

## Results

### Participants

Twenty-eight young adults participated (11 females). The average age, height, weight, and body mass index were 22.4 ± 5.2 years, 1.69 ± 0.07 meters, 69.0 ± 10.2 kg, and 23.9 ± 2.7 kg/m^2^, respectively.

### GI

Table 1 presents the GI variables. We excluded 9 participants due to protocol violations (e.g., fidgeting their arms). We found significant differences following dual task effects in GI in total GI duration, all the phase durations, CoM displacement, GRF, and COFP displacement.

**Table 1:** GI variables of single-task versus dual-task.

Variables	Single-Task	Dual-Task	P-Value	Effect Size
**Phase durations (Seconds)**				
Total GI duration	1.37 ± 0.19	1.48 ± 0.23	0.005*	0.44
Phase 1: Weight shift	0.35 ± 0.15	0.32 ± 0.09	< 0.001*	0.86
Phase 2: Pre-swing of stepping limb	0.38 ± 0.12	0.43 ± 0.14	0.017*	0.37
Phase 3: Pre-swing of stance limb	0.64 ± 0.07	0.73 ± .11	0.001*	0.81
**Phase durations (Normalized; %)**				
Weight shift	24.8 ± 10.7	22.8 ± 7.0	0.017*	0.17
Pre-swing of stepping limb	26.9 ± 8.6	30.6 ± 10.1	0.001*	0.37
Pre-swing of stance limb	45.2 ± 5.2	51.9 ± 8.1	<0.001*	0.72
**CoM Displacement (centimeters)**				
Mediolateral	6.2 ± 2.0	7.2 ± 2.2	0.001*	0.48
Vertical	2.4 ± 0. 9	1.7 ± 0. 8	0.001*	0.65
**Vertical GRF (%BW)**				
Weight shift stepping max	14.4 ± 7.9	17.3 ± 6.0	0.004*	0.31
Weight shift stance min	−15.5 ± 8.4	−18.2 ± 6.4	0.007*	0.28
Stance loading	66.6 ± 12.6	68.2 ± 9.0	0.352	0.12
**Anterior-Posterior GRF (%BW)**				
Stance Propulsion	17.8 ± 4.9	13.7 ± 5.1	0.001*	0.92
Stepping Propulsion	3.1 ± 1.9	2.4 ± 1.5	0.117	0.28
**Mediolateral GRF (%BW)**				
Stepping Lateral	3.7 ± 1.9	4.1 ± 1.6	0.102	0.16
Stance Medial	−2.0 ± 1.3	−2.2 ± 1.3	0.03*	0.15
Stance Lateral	6.8 ± 2.1	6.4 ± 1.6	0.232	0.19
**COFP Displacements (centimeters)**				
Stance Anterior	18.4 ± 3.1	19.4 ± 8.7	0.392	0.13
Stance Posterior	3.9 ± 1.7	3.2 ± 2.0	0.01*	0.36
Stepping Anterior	13.2 ± 7.4	13.5 ± 7.3	0.448	0.04
Stepping Posterior	2.6 ± 1.4	2.3 ± 1.2	0.171	0.18

Note: Asterisks indicate p < 0.05. Effect sizes were calculated as Cohen’s d.

**Fig 5:**
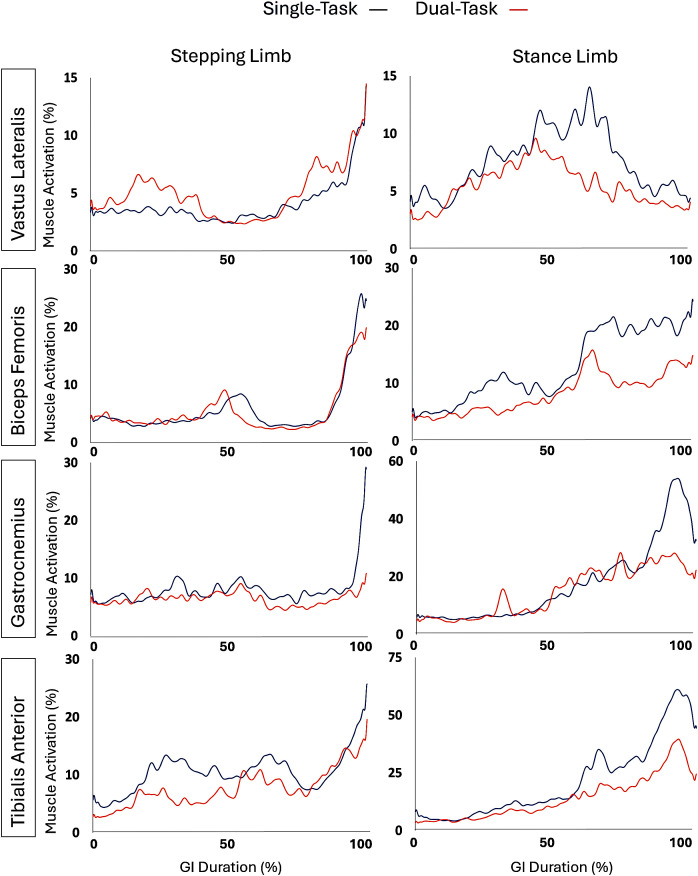
EMG data during GI. The EMG data was averaged across all participants.

The pattern of muscle activation in the vastus lateralis, biceps femoris, gastrocnemius, and tibialis anterior in the stepping and stance limbs during the entire GI is shown in Fig 5.

Results for muscle activation are shown in Table 2. Overall, we found a significant difference during the pre-swing phase of the stepping and stance limbs, particularly in the biceps femoris and tibialis anterior muscles.

**Table 2:** Magnitudes (% MVC) of the maximum EMG in each GI phase and entire GI.

Muscle Group in Phase	Single-Task	Dual-Task	P-Value	Effect Size
**Weight Shift Phase**				
Biceps Femoris (stepping limb)	3.6 ± 2.0	3.8 ± 2.6	0.843	0.07
Biceps Femoris (stance limb)	5.4 ± 4.9	3.9 ± 3.9	0.055	0.27
Gastrocnemius (stepping limb)	4.9 ± 3.6	6.2 ± 3.5	0.294	0.26
Gastrocnemius (stance limb)	5.8 ± 4.6	5.6 ± 6.6	0.10	0.02
Tibialis Anterior (stepping limb)	5.4 ± 6.5	4.3 ± 3.7	0.144	0.14
Tibialis Anterior (stance limb)	5.0 ± 4.9	4.2 ± 2.9	0.244	0.17
Vastus Lateralis (stepping limb)	3.4 ± 3.3	3.7 ± 2.5	0.636	0.08
Vastus Lateralis (stance limb)	5.0 ± 4.8	3.8 ± 3.0	0.191	0.21
**Pre-Swing of Stepping Limb Phase**				
Biceps Femoris (stepping limb)	5.3 ± 4.9	5.0 ± 4.4	0.884	0.04
Biceps Femoris (stance limb)	10.0 ± 12.0	6.4 ± 4.9	0.132	0.28
Gastrocnemius (stepping limb)	7.3 ± 3.7	6.8 ± 7.1	0.050	0.07
Gastrocnemius (stance limb)	8.2 ± 8.5	9.4 ± 12.2	0.831	0.07
Tibialis Anterior (stepping limb)	9.6 ± 11.3	6.1 ± 4.9	0.044*	0.29
Tibialis Anterior (stance limb)	11.9 ± 13.8	9.0 ± 7.3	0.186	0.19
Vastus Lateralis (stepping limb)	3.2 ± 2.8	3.5 ± 2.5	0.577	0.09
Vastus Lateralis (stance limb)	9.1 ± 9.8	7.3 ± 6.2	0.281	0.17
**Pre-Swing of Stance Limb Phase**				
Biceps Femoris (stepping limb)	7.3 ± 5.0	6.1 ± 4.7	0.129	0.19
Biceps Femoris (stance limb)	18.6 ± 15.4	11.4 ± 8.1	0.023*	0.58
Gastrocnemius (stepping limb)	7.5 ± 10.1	6.1 ± 2.9	0.419	0.14
Gastrocnemius (stance limb)	28.4 ± 19.0	22.7 ± 17.9	0.160	0.22
Tibialis Anterior (stepping limb)	11.9 ± 5.5	10.4 ± 4.0	0.037*	0.25
Tibialis Anterior (stance limb)	23.1 ± 17.7	22.5 ± 19.3	0.845	0.03
Vastus Lateralis (stepping limb)	4.6 ± 3.4	5.0 ± 2.7	0.618	0.08
Vastus Lateralis (stance limb)	7.8 ± 6.5	5.1 ± 3.8	0.10	0.41
**Total GI**				
Biceps Femoris (stepping limb)	5.8 ± 3.5	5.4 ± 3.6	0.354	0.10
Biceps Femoris (stance limb)	12.9 ± 9.4	8.3 ± 5.5	0.011*	0.56
Gastrocnemius (stepping limb)	6.6 ± 6.6	6.4 ± 2.9	0.890	0.02
Gastrocnemius (stance limb)	16.9 ± 10.2	14.9 ± 12.1	0.427	0.13
Tibialis Anterior (stepping limb)	9.6 ± 6.0	7.7 ± 3.6	0.036*	0.27
Tibialis Anterior (stance limb)	18.9 ± 22.0	14.0 ± 10.8	0.098	0.21
Vastus Lateralis (stepping limb)	4.0 ± 2.4	4.1 ± 2.6	0.851	0.03
Vastus Lateralis (stance limb)	7.5 ± 6.3	5.5 ± 4.0	0.276	0.30

Note: Asterisks indicate p < 0.05. Effect sizes were calculated as Cohen’s d.

### STW

For STW results, data of 7 participants were excluded due to protocol violations that were not caught during data collection (e.g., shaking legs slightly). The STW kinematic variables are shown in Table 3. Overall, dual-tasking significantly increased the durations for phases 3 and 4, and total phase duration, and hesitation, and decreased the initial step length (all p < 0.05).

**Table 3:** STW variables of single-task versus dual-task.

Outcome	Single-Task	Dual-Task	P-Value	Effect Size
**Phase Duration (seconds)**				
Total Phase	1.84 ± 0.27	2.02 ± 0.35	0.001*	0.47
Phase 1: Flexion Momentum	0.85 ± 0.17	0.86 ± 0.22	0.809	0.06
Phase 2: Extension	0.38 ± 0.08	0.39 ± 0.06	0.210	0.17
Phase 3: Unloading	0.41 ± 0.13	0.51 ± 0.20	0.004*	0.43
Phase 4: Stance	0.57 ± 0.06	0.65 ± 0.11	0.001*	0.67
**Peak COM Velocity (m/s)**				
Mediolateral	0.19 ± 0.09	0.21 ± 0.10	0.478	0.06
Anterior-Posterior	0.51 ± 0.13	0.48 ± 0.12	0.369	0.28
Velocity Vertical	0.68 ± 0.12	0.65 ± 0.09	0.447	0.23
**Peak COM Displacement (meters)**				
Mediolateral	0.07 ± 0.04	0.08 ± 0.05	0.397	0.15
Anterior-Posterior	0.61 ± 0.10	0.60 ± 0.10	0.45	0.10
Vertical	0.25 ± 0.03	0.26 ± 0.02	0.052	0.45
**Hesitation (%)**	34 ± 20	43 ± 18	0.041*	0.29
**Absolute Velocity Drop (m/s)**	0.18 ± 0.11	0.21 ± 0.10	0.061	0.26
**Initial Step Length (meters)**	0.64 ± 0.10	0.58 ± 0.12	0.004*	0.45

Note: Asterisks indicate p < 0.05. Effect sizes were calculated as Cohen’s d.

**Fig 6:**
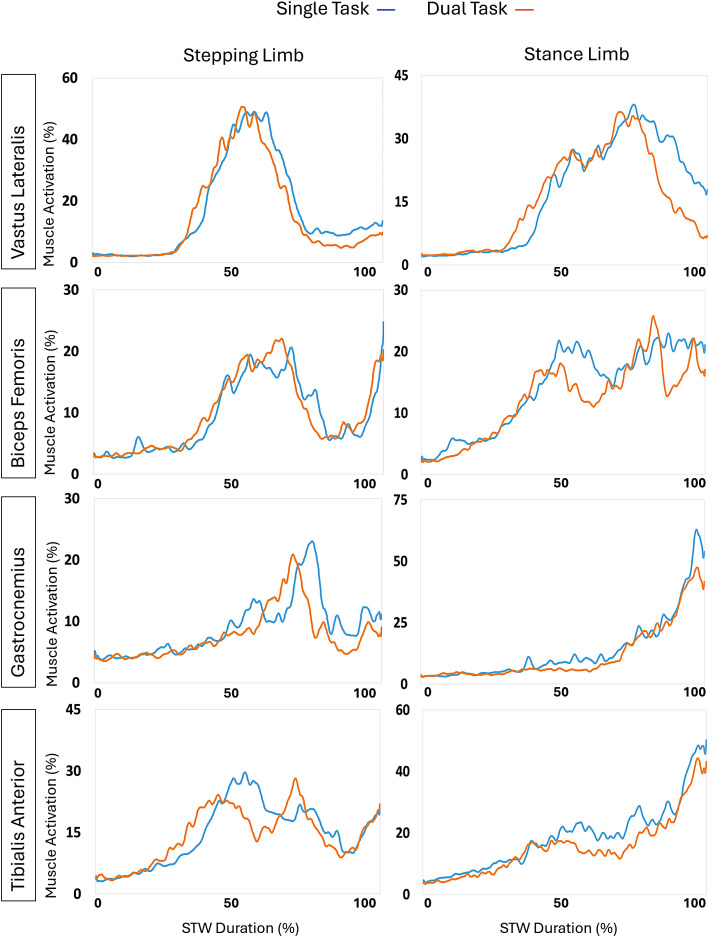
EMG data during STW. The EMG data was averaged across all participants.

Fig 6 displays the different muscle activations across the STW duration. The EMG data was averaged across all participants.

Table 4 shows the EMG outcomes for STW. During phase 1, dual-tasking significantly decreased the activation of the biceps femoris in the stance limb (p = 0.007) with a medium effect. During phase 2, dual-tasking significantly decreased the activation of the gastrocnemius in both limbs (all p < 0.05) with medium effects, and similar trends were found in phase 4.

**Table 4:** Magnitudes (% MVC) of the maximum EMG in each STW phase and entire STW.

Muscle	Single-Task	Dual-Task	P-Value	Effect Size
**Phase 1: Flexion Momentum Phase**				
Biceps Femoris (stepping limb)	4.7 ± 5.4	4.1 ± 3.6	0.973	0.14
Biceps Femoris (stance limb)	7.5 ± 4.9	5.8 ± 6.2	0.007*	0.23
Gastrocnemius (stepping limb)	5.2 ± 3.0	4.3 ± 2.4	0.433	0.28
Gastrocnemius (stance limb)	4.4 ± 2.4	4.3 ± 2.2	0.913	0.05
Tibialis Anterior (stepping limb)	9.2 ± 5.9	9.3 ± 6.8	0.926	0.01
Tibialis Anterior (stance limb)	9.5 ± 9.6	8.3 ± 6.5	0.853	0.10
Vastus Lateralis (stepping limb)	6.2 ± 4.5	5.6 ± 3.2	0.636	0.11
Vastus Lateralis (stance limb)	4.7 ± 3.4	4.5 ± 3.1	0.726	0.05
**Phase 2: Extension Phase**				
Biceps Femoris (stepping limb)	17.5 ± 14.7	18.8 ± 13.2	0.17	0.09
Biceps Femoris (stance limb)	21.2 ± 12.9	17.8 ± 12.4	0.174	0.21
Gastrocnemius (stepping limb)	12.2 ± 7.9	9.1 ± 7.5	0.036*	0.32
Gastrocnemius (stance limb)	11.2 ± 10.0	6.9 ± 3.5	0.048*	0.42
Tibialis Anterior (stepping limb)	26.0 ± 15.8	24.0 ± 15.0	0.432	0.10
Tibialis Anterior (stance limb)	22.6 ± 19.4	18.6 ± 15.2	0.424	0.15
Vastus Lateralis (stepping limb)	47.1 ± 18.2	48.5 ± 16.5	0.673	0.07
Vastus Lateralis (stance limb)	28.6 ± 22.0	28.3 ± 16.9	0.759	0.01
**Phase 3: Unloading Phase**				
Biceps Femoris (stepping limb)	15.4 ± 12.0	18.9 ± 12.8	0.039*	0.24
Biceps Femoris (stance limb)	19.6 ± 12.8	15.1 ± 10.8	0.086	0.34
Gastrocnemius (stepping limb)	11.0 ± 6.9	10.7 ± 10.4	0.103	0.02
Gastrocnemius (stance limb)	10.4 ± 11.1	6.6 ± 3.5	0.06	0.35
Tibialis Anterior (stepping limb)	23.1 ± 13.4	19.3 ± 11.0	0.198	0.23
Tibialis Anterior (stance limb)	20.6 ± 19.1	16.6 ± 13.9	0.663	0.17
Vastus Lateralis (stepping limb)	42.2 ± 20.0	42.0 ± 18.5	0.947	0.01
Vastus Lateralis (stance limb)	25.8 ± 21.3	25.8 ± 17.2	0.628	0
**Phase 4: Stance Phase**				
Biceps Femoris (stepping limb)	10.2 ± 6.9	11.3 ± 13.9	0.99	0.09
Biceps Femoris (stance limb)	19.1 ± 13.6	15.1 ± 11.1	0.054	0.26
Gastrocnemius (stepping limb)	13.2 ± 10.4	8.6 ± 7.9	0.004*	0.52
Gastrocnemius (stance limb)	29.2 ± 17.3	25.6 ± 10.2	0.554	0.20
Tibialis Anterior (stepping limb)	16.8 ± 12.5	16.6 ± 8.5	0.885	0.01
Tibialis Anterior (stance limb)	30.4 ± 13.4	25.0 ± 10.6	0.038*	0.32
Vastus Lateralis (stepping limb)	11.3 ± 10.3	7.3 ± 7.3	0.003*	0.38
Vastus Lateralis (stance limb)	28.5 ± 19.7	19.9 ± 13.2	0.073	0.38
**Total STW**				
Biceps Femoris (stepping limb)	9.5 ± 7.6	9.9 ± 6.1	0.279	0.05
Biceps Femoris (stance limb)	14.2 ± 7.2	12.3 ± 9.9	0.264	0.16
Gastrocnemius (stepping limb)	9.0 ± 5.0	7.1 ± 4.8	0.032*	0.37
Gastrocnemius (stance limb)	14.0 ± 7.8	12.0 ± 4.4	0.557	0.28
Tibialis Anterior (stepping limb)	14.5 ± 7.6	14.2 ± 6.8	0.89	0.03
Tibialis Anterior (stance limb)	18.7 ± 10.8	15.7 ± 6.6	0.404	0.24
Vastus Lateralis (stepping limb)	16.6 ± 6.3	15.2 ± 5.0	0.218	0.19
Vastus Lateralis (stance limb)	17.0 ± 10.3	15.1 ± 8.1	0.309	0.15

Note: Asterisks indicate p < 0.05. Effect sizes were calculated as Cohen’s d.

## Discussion

We investigated changes of GI and STW in response to dual-tasking in young adults. Significant changes were observed in all kinematic, kinetic and muscle activity patterns during GI and STW. All phases of GI including weight shift, pre-swing of the stepping limb, pre-swing of the stance limb, and total duration were found to be significantly affected. While the unloading, stance, and total STW phase was found to be significantly affected. Among these phases weight shift phase exhibited a decrease, while all the other temporal durations displayed increased durations in response to dual task. These findings align with literature that investigated dual-task effects on anticipatory postural adjustment where they found increased durations in healthy subjects [10] and in studies investigating aging populations and frailty [23].

The role of compensatory behaviors helps explain the differences in responses observed between our study and others, suggesting that these behaviors play a key role in how individuals manage the challenges posed by dual-task situations. Previous literature suggests that GI is influenced not only by task instructions and environmental factors [24], but also the task priority [25]. Younger people tend to manage complex cognitive-motor tasks better than older adults [26]. Some individuals may adopt a “posture-second” strategy, focusing more on cognitive tasks than motor tasks, which can lead to biomechanical adjustments [27]. For older adults and those with Parkinson’s disease, this task prioritization may differ, leading to different responses. The longer durations observed in GI and STW in our study, and similar studies with healthy young adults, likely indicate a compensation strategy for handling the increased complexity of dual tasks.

When analyzing temporal parameters, it is important to connect them with kinetic responses during GI. A previous study reported that GRF during the weight shift phase, were lower in young adults compared to older adults, and the thrust phase lasted longer in the elderly [28]. In our study, we observed a shorter weight shift phase, which could be due to differences in task prioritization between younger and older adults. Increased durations or force magnitudes in young adults might indicate instability in the movement but also suggest that they are compensating effectively. Young adults are likely to adopt a posture-secondary strategy, as their risk of falling is lower, whereas older adults may prioritize stability over cognitive tasks in complex movements.

During GI, we observed a reduction in vertical and anterior-posterior GRF related to stance propulsion. In STW, increased hesitation led to reduced forward momentum and less fluidity, reflected in shorter step length. These results align with previous studies, supporting the idea that dual-tasking demands greater postural stability. While COFP path length typically increases with conditions like obesity due to compensatory behavior, our study showed decreased posterior displacement and shorter path lengths, despite increased weight shifts. This suggests that dual-task conditions may constrain weight-shifting ability, leading to a less efficient response.

Our EMG results indicate that during dual-task scenarios, individuals tend to adopt compensatory strategies that either impede forward anterior-posterior movement or enhance balance control through increased activation of muscles associated with stability. Waldon et al. (2023) emphasizes the role of the tibialis anterior muscle in clearing the foot off the ground during the initial swing phase [29]. Fundamentally, the forward momentum of the stepping limb cannot begin until the limb is cleared from the ground. Our results align with these findings, revealing reduced tibialis anterior activation during the pre-swing phase of the stepping limb during GI. This decreased activation suggests that dual task conditions do impair the forward momentum of the stepping limb, supporting the notion that dual task conditions can inhibit motor performance in some manner.

During STW, the pattern was slightly different from GI. In particular, the gastrocnemius muscle, an ankle plantar flexion, emerged as the most crucial muscle for adjusting to dual-tasking during STW. Given the significant difference in hesitation between single-task and dual-task conditions, the gastrocnemius plays a key role in maintaining balance during STW. This may indicate that the gastrocnemius is essential for stabilizing the body as it shifts from sitting to walking, helping to manage the added complexity of a cognitive task. The importance of this muscle in STW might also reflect its function in generating the push needed to propel the body forward, which could be particularly sensitive to dual-task demands. In contrast, during GI, other muscles may share the load more evenly. The findings suggest that effective balance control in STW, especially under dual-task conditions, depends heavily on gastrocnemius activation.

Limitations should be acknowledged. First, the cognitive dual-task employed in our study (i.e., serial subtraction by 3), was relatively simple. Although even this low cognitive load produced significant effects on both GI and STW, future studies should examine a broader range of cognitive tasks to better understand differential impacts on transitional movements. Second, while characterizing healthy young adults provides important benchmark data, the generalizability of our findings to other populations remains limited. Future research should assess these movement patterns in diverse age groups and in individuals with mobility impairments (e.g., those at high risk of falls). Finally, since the study was conducted in a controlled laboratory setting, it is important to validate these findings in more ecologically valid environments that better simulate daily-life scenarios.

Nevertheless, our findings significantly enhance our understanding of transitional movements and could lead to better understanding of balance control mechanisms for challenging motor tasks. Additionally, the findings provide useful baseline data for comparison with older adults or those at risk of falls, and highlight the importance of considering cognitive factors in movement assessments.
